# Systemic Factors Associated With Severe Dysphagia 28 Days After Extubation in ICU Patients With Post-extubation Dysphagia

**DOI:** 10.7759/cureus.110112

**Published:** 2026-06-02

**Authors:** Ayaka Fujita, Ippei Tamura, Eiji Hashiba, Seiko Hasegawa, Naoya Matsuda, Eiichi Tsuda

**Affiliations:** 1 Rehabilitation Medicine, Hirosaki University Hospital, Hirosaki, JPN; 2 Rehabilitation, Hirosaki University Hospital, Hirosaki, JPN; 3 Anesthesiology, Hirosaki University Hospital, Hirosaki, JPN; 4 Emergency and Disaster Medicine, Hirosaki University, Hirosaki, JPN; 5 Rehabilitation Medicine, Hirosaki University Graduate School of Medicine, Hirosaki, JPN

**Keywords:** deglutition disorders, intensive care unit, mechanical ventilation, respiratory rate, swallowing rehabilitation

## Abstract

Objectives

To identify clinical and respiratory factors associated with severe dysphagia at 28 days after extubation, defined as a Food Intake Level Scale (FILS) score of 1-3 with continued dependence on enteral nutrition, among critically ill patients with post-extubation dysphagia (PED).

Methods

We retrospectively reviewed intensive care unit (ICU) patients with PED who had been able to eat orally before admission and subsequently received swallowing rehabilitation by speech-language pathologists at a single center between April 2021 and September 2023. Patients with a FILS score of 1-3 at 28 days after extubation who still required enteral nutrition were classified as having severe dysphagia. Patients were classified into a severe dysphagia group (FILS score 1-3; n = 16) and a non-severe dysphagia group (FILS score 4-10; n = 39). We examined factors related to severe dysphagia using univariable and multivariable analyses.

Results

Respiratory rate at the initial post-extubation swallowing assessment was higher in the severe dysphagia group than in the non-severe dysphagia group, whereas other vital signs and laboratory findings were similar. In multivariable analysis, respiratory rate (odds ratio (OR): 1.72; 95% confidence interval (CI): 1.31-2.68; P < 0.0001) and the duration of intubation (OR: 1.21; 95% CI: 1.00-1.52; P = 0.0497) were independently associated with severe dysphagia at 28 days after extubation.

Conclusions

Higher respiratory rate at the initial swallowing assessment and longer duration of intubation were independently associated with severe dysphagia 28 days after extubation in ICU patients with PED. These findings may help clinicians identify patients at risk of prolonged dysphagia after extubation.

## Introduction

Post-extubation dysphagia (PED) frequently occurs in critically ill patients receiving mechanical ventilation and is associated with prolonged hospitalization and poor clinical outcomes [1-3]. Early swallowing assessment and rehabilitation are important for safe resumption of oral intake after extubation [2,4].

Previous studies have suggested that delayed swallowing rehabilitation after extubation may lead to worse outcomes, including aspiration pneumonia and persistent dysphagia [4]. Among critically ill patients, swallowing assessment and rehabilitation are commonly performed by speech-language pathologists (SLPs) [5]. Basic readiness criteria for swallowing assessment, such as adequate alertness and the ability to maintain an upright position, have also been proposed [2]. However, in clinical practice, some patients who meet these criteria may still be poor candidates for immediate oral intake training.

Starting oral intake too early after extubation may increase the risk of aspiration and worsen respiratory status, whereas delaying swallowing rehabilitation too long may prolong dysphagia and dependence on enteral nutrition. Despite this clinical dilemma, bedside indicators that may help determine readiness for oral intake after extubation remain insufficiently described.

Therefore, this study examined whether clinical and respiratory factors present at the initial swallowing assessment after extubation were associated with severe dysphagia (SD) at 28 days in intensive care unit (ICU) patients with PED. SD was defined as continued dependence on enteral nutrition at 28 days after extubation. We hypothesized that these early bedside factors would be associated with later SD.

## Materials and methods

Study design and patients

This retrospective cohort study evaluated PED in relation to vital signs and illness severity in ICU patients. This study received approval from the Institutional Ethics Committee (approval no. 2023-147-1) and was conducted in accordance with the Declaration of Helsinki. Because of the retrospective design and minimal risk, the requirement for informed consent was waived, and an opt-out approach was used. This study adhered to the Strengthening the Reporting of Observational Studies in Epidemiology (STROBE) guidelines.

**Figure 1:**
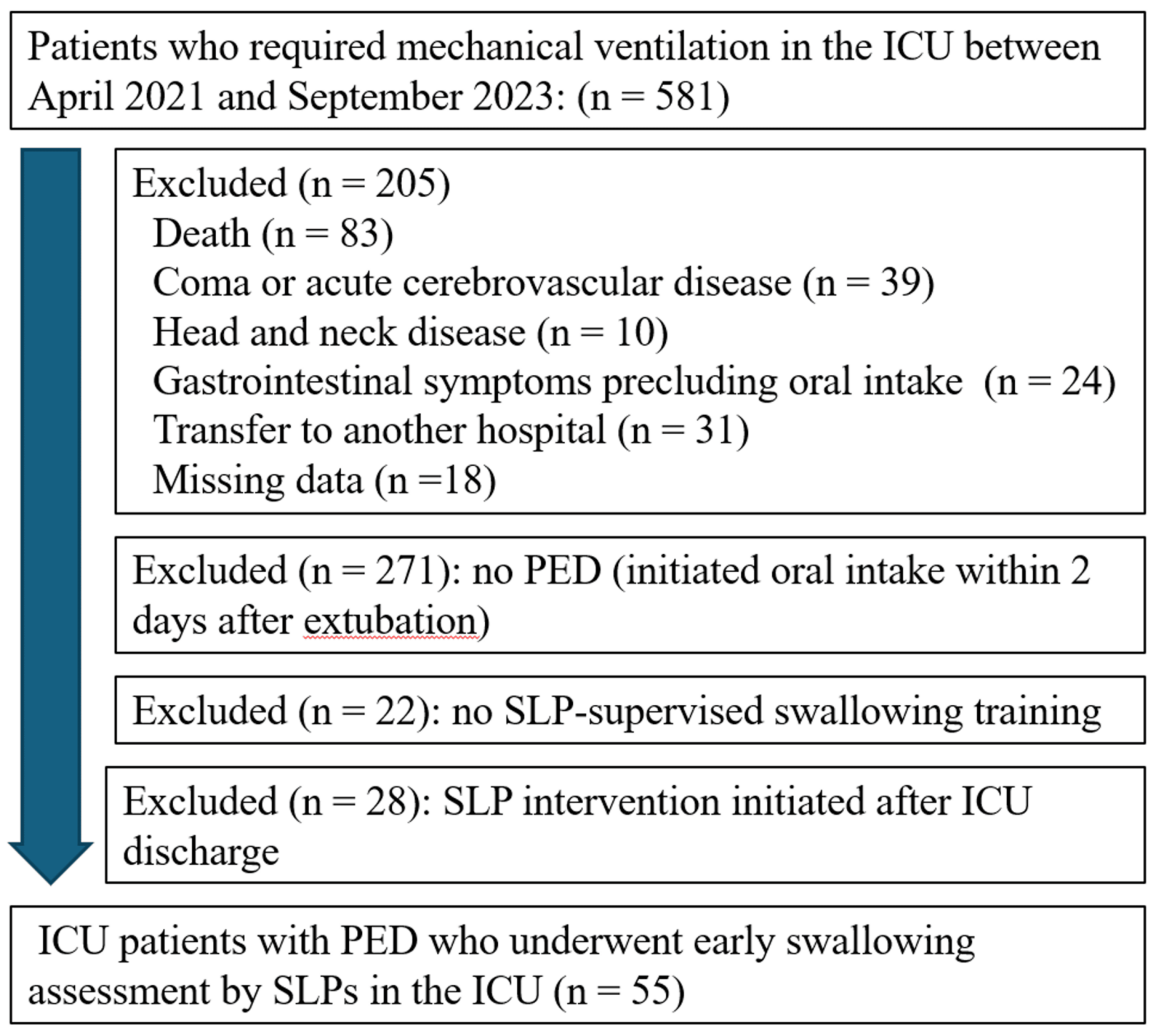
Flow diagram illustrating patient selection for the study. Patients with PED who underwent early swallowing assessment by SLPs in the ICU were included in the final analysis. ICU: intensive care unit; PED: post-extubation dysphagia; SLP: speech-language pathologist

We consecutively screened 581 patients who underwent extubation after at least 24 hours of mechanical ventilation in the general or emergency ICU between April 2021 and September 2023. All patients had normal oral intake before ICU admission. Among these patients, 55 patients with PED who remained conscious and underwent early swallowing assessment by SLPs in the ICU were included (Figure 1).

Patients were referred to the rehabilitation team when they failed a nurse-administered water-swallow or food test or were unable to initiate oral intake within 48 hours after extubation despite adequate wakefulness. We excluded patients with head and neck disorders, acute cerebrovascular disease as the primary reason for ICU admission, gastrointestinal symptoms preventing oral intake, persistent coma, death during the acute phase, or transfer to another hospital immediately before or after extubation. Patients who underwent swallowing rehabilitation only after ICU discharge were also excluded. Patients with missing data for key variables, including respiratory rate (RR) or laboratory data, were additionally excluded.

All patients underwent swallowing assessment by an SLP and a rehabilitation physician in the ICU. Swallowing rehabilitation was generally performed for 20 minutes per session, five times per week during ICU stay. After ICU discharge, rehabilitation frequency was adjusted according to patient condition and ward management policy.

We retrospectively reviewed clinical information from the medical records, including patient characteristics, oxygen therapy, vital signs, and laboratory data at the initial swallowing assessment.

Vital signs and laboratory data

The vital signs assessed in the ICU were mean arterial pressure (MAP), heart rate, RR, and body temperature. The Glasgow Coma Scale (GCS) was used to assess consciousness level [6]. Laboratory data obtained during ICU stay included arterial partial pressure of oxygen (PaO_2_), platelet count, serum creatinine, and total bilirubin. These variables were used to calculate Sequential Organ Failure Assessment (SOFA) [7] score and the PaO_2_/FiO_2_ (P/F) ratio as indicators of illness severity and oxygenation.

Swallowing assessment and rehabilitation

All patients were evaluated using the Modified Water Swallowing Test (MWST) and a standardized food test in an appropriate head, neck, and trunk position to reduce aspiration risk. The MWST is a validated bedside dysphagia screening tool widely used in Japan and was performed using a 3-mL water bolus with a five-point scoring system [8].

The food test used a 4-g jelly bolus and the same scoring framework as the MWST [8]. Dysphagia was diagnosed when at least 25% of the jelly remained in the mouth after swallowing.

When clinically indicated, swallowing function was evaluated using fiberoptic endoscopic evaluation of swallowing (FEES) [9]. Food-based swallowing training was not initiated when FEES showed airway invasion below the vocal folds or when aspiration was suspected during bedside swallowing tests based on clinical signs such as coughing, wet voice, hoarseness, or gurgling breath sounds [9].

Based on the initial swallowing assessment, rehabilitation was categorized as non-food training or food-based training. Non-food training included exercises targeting tongue, pharyngeal, and laryngeal muscle function, voice training, and respiratory-swallow coordination. Food-based training included posture adjustment, modification of food texture and bolus volume, and compensatory swallowing strategies [10]. Reassessment was performed during rehabilitation, and training content was adjusted according to patient condition.

Definition of severe dysphagia (SD)

The Food Intake Level Scale (FILS) [11] was used to evaluate swallowing function and oral intake status on a 10-point scale. Patients with a FILS score of 1-3 at 28 days after extubation were classified as having SD, indicating continued dependence on enteral nutrition. Patients were therefore classified into the SD group (FILS 1-3) and the non-severe dysphagia (NSD) group (FILS 4-10).

FILS scores at 28 days were retrospectively determined from medical records based on dietary status, amount and type of oral intake, and use of alternative nutrition. In patients transferred to another hospital before day 28, nutritional status at 28 days after extubation was confirmed by telephone with the receiving institution.

Statistical analysis

Statistical analyses were performed using JMP Student Edition version 18.2.0 (SAS Institute Inc., Cary, NC, USA). Continuous variables are presented as median and interquartile range, and categorical variables as number and percentage. The Shapiro-Wilk test was used to assess data distribution.

Continuous variables were compared using the Mann-Whitney U test. Categorical variables were analyzed using Fisher’s exact test or the chi-square test, as appropriate. All statistical tests were two-sided, and P values < 0.05 were considered statistically significant.

Multivariable logistic regression analysis was performed to examine factors associated with SD at 28 days after extubation. Variables showing a P value < 0.10 in the univariable analyses were included in the multivariable model. Because the SOFA score was strongly correlated with GCS and did not meet the predefined inclusion criterion, it was not included in the final multivariable model. GCS was retained because it was considered more clinically relevant to swallowing readiness. Owing to the limited sample size and number of events, the multivariable analysis was considered exploratory.

Because this study used a retrospective exploratory design at a single institution, a formal sample size calculation was not carried out before the analysis. We included all eligible consecutive patients treated during the study period. As a result, the study size depended on the number of patients who satisfied the inclusion criteria. To reduce the risk of overfitting, only a limited number of clinically relevant variables were included in the multivariable model.

## Results

The clinical characteristics of the 55 patients are presented in Table 1. SD at 28 days after extubation was observed in 16 patients (29.1%). Patients were categorized into the SD group (FILS score 1-3; n = 16) and the NSD group (FILS score 4-10; n = 39).

**Table 1:** Clinical characteristics of patients with severe and non-severe dysphagia. Comparisons between groups were performed using the Mann-Whitney U test for continuous variables and the chi-square test for categorical variables. Data are presented as n (%) or median (IQR). * P-value < 0.05 was considered statistically significant. BMI: body mass index; SOFA: Sequential Organ Failure Assessment; FILS: Food Intake Level Scale

Variables	Severe dysphagia (n = 16)	Non-severe dysphagia (n = 39)	Test statistic	p-value
Age, years	66.5 (57-73)	70 (57-74)	Z = -0.83	0.41
Female sex, n (%)	5 (31.3)	17 (43.6)	χ^2^ = 0.73	0.39
BMI, kg/m^2^	22.9 (21.4-26.7)	24.3 (20.1-27.1)	Z = -0.19	0.85
Hypertension, n (%)	5 (31.3)	11 (28.2)	χ^2^ = 0.05	0.82
History of stroke, n (%)	5 (31.3)	6 (15.4)	χ^2^ = 1.68	0.19
Heart disease, n (%)	6 (37.5)	10 (25.6)	χ^2^ = 0.75	0.38
Respiratory disease, n (%)	1 (6.3)	2 (5.1)	Fisher’s exact test	0.87
Diabetes, n (%)	3 (18.8)	9 (21.8)	χ^2^ = 0.13	0.72
Duration of intubation (days)	10 (6.25-12)	5 (3-9)	Z = 2.76	0.006*
Interval from extubation to swallowing assessment (days)	3.5 (1-11)	1 (1-3)	Z = 2.06	0.040*
SOFA score at the initial assessment	5 (3.25-6.75)	4 (2-6)	Z = 1.33	0.18
FILS at the initial assessment	1 (1-1.75)	3 (2-4)	Z = -4.17	<0.0001*
Duration of hospitalization (days)	46 (24-79)	28 (17-46)	Z = 1.71	0.09

The SD group had a longer duration of intubation than the NSD group (10 vs. 5 days; P = 0.006). The interval from extubation to the initial swallowing assessment was also longer in the SD group (3.5 vs. 1.0 days; P = 0.040). The SD group also showed a lower initial FILS score than the NSD group (1 vs. 3; P < 0.0001). Age, sex, body mass index, medical history, SOFA score at the initial assessment, and duration of hospitalization were similar between the groups (Table 2).

At the initial swallowing assessment, the RR was higher in the SD group than in the NSD group (28.5 vs 22.0 breaths/min; P < 0.0001). The proportion of patients who achieved FILS ≥ 3 in the ICU was lower in the SD group than in the NSD group (6.25% vs 69.2%; P < 0.0001). Oxygen therapy, P/F ratio, heart rate, MAP, GCS score, body temperature, and laboratory findings did not differ significantly between the groups (Table 2).

**Table 2:** Oxygen therapy, vital signs, and laboratory data at the initial swallowing assessment in patients with severe and non-severe dysphagia. Comparisons between groups were performed using the Mann-Whitney U test for continuous variables and the chi-square test or Fisher’s exact test for categorical variables. Data are presented as n (%) or median (IQR). * P-value < 0.05 was considered statistically significant. P/F: PaO_2_/FiO_2_ ratio; FILS: Food Intake Level Scale; GCS: Glasgow Coma Scale; mmHg: millimeters of mercury

Variables	Severe dysphagia (n = 16)	Non-severe dysphagia (n = 39)	Test statistic	p-value	
Oxygen therapy at initial assessment	N/A	N/A	Fisher’s exact test	0.23	
Face mask, n (%)	1 (6.25)	7 (18.0)	N/A	N/A	
Nasal cannula, n (%)	5 (31.3)	17 (43.6)	N/A	N/A	
High-flow nasal cannula, n (%)	4 (25.0)	7 (18.0)	N/A	N/A	
Tracheostomy mask, n (%)	3 (18.8)	1 (2.6)	N/A	N/A	
Mechanical ventilation via tracheostomy, n (%)	2 (12.5)	2 (5.1)	N/A	N/A	
None, n (%)	1 (6.25)	5 (12.8)	N/A	N/A	
Vital signs and laboratory data at initial swallowing assessment					
P/F ratio	269 (193-336)	296 (230-354)	Z = -0.91	0.36	
Respiratory rate, breaths per minute	28.5 (27-33)	22 (18-25)	Z = 4.99	<0.0001*	
Mean arterial pressure, mmHg	77 (66.5-90)	88 (66-92.5)	Z =-1.35	0.18	
Heart rate, beats per minute	99 (80.8-108.8)	91 (76-99)	Z = 1.55	0.12	
GCS	14 (13.25-14)	14 (14-15)	Z = -1.68	0.09	
Temperature, ℃	37.0 (36.4-37.4)	37.2 (36.8-37.4)	Z = -1.50	0.13	
Serum total bilirubin, mg/dL	1.2 (0.4-1.8)	0.7 (0.5-1.3)	Z = 0.50	0.61	
Platelet count (×10^4^/µL)	15.8 (12.3-28.0)	18.8 (13.5-26.0)	Z =-0.44	0.66	
Serum creatinine, mg/dL	1.04 (0.56-2.30)	0.76 (0.57-1.91)	Z = 0.36	0.71	
FILS ≥ 3 in ICU, n (%)	1 (6.25)	27 (69.2)	Fisher’s exact test	<0.0001*	

In the multivariable logistic regression model, longer duration of intubation and higher RR were independently associated with SD 28 days after extubation. Higher RR was associated with increased odds of SD (OR 1.72; 95% CI 1.31-2.68; P < 0.0001). Longer duration of intubation was also independently associated with SD (OR 1.21; 95% CI 1.00-1.52; P = 0.0497). GCS did not show an independent relationship with SD (Table 3).

**Table 3:** Multivariable logistic regression analysis of factors associated with severe dysphagia 28 days after extubation. ORs and 95% CIs are shown for each 1-unit increase in the continuous variables. * P-value < 0.05 was considered statistically significant. GCS: Glasgow Coma Scale; OR: odds ratio; CI: confidence interval

	OR	95% CI	P-value
Duration of intubation (days)	1.21	1.00-1.52	0.0497*
GCS	0.30	0.06-1.15	0.080
Respiratory rate (breaths/min)	1.72	1.31-2.68	<0.0001*

## Discussion

In this study, higher RR and longer intubation duration were independently related to SD 28 days after extubation in ICU patients with PED. These findings suggest a possible relationship between early respiratory status and later swallowing outcomes after extubation.

Previous studies have reported several factors associated with PED in ICU patients, including age, duration of intubation, illness severity, and tracheostomy [12]. In the present study, age, comorbidities, and baseline SOFA score were not significantly different between the SD and NSD groups. Duration of intubation and RR were independently associated with SD in the multivariable model. Longer intubation may reflect prolonged critical illness and greater exposure to factors that impair swallowing function. Longer duration of intubation may contribute to swallowing dysfunction through disuse of swallowing-related muscles, laryngeal injury, or prolonged critical illness.

Early swallowing assessment after extubation remains clinically challenging. Early oral intake may increase aspiration risk and worsen respiratory status, whereas delayed intervention may contribute to prolonged dysphagia [4]. In our cohort, the median RR in the SD group was 28.5 breaths/min. Previous studies involving patients with acute hypoxemic respiratory failure have suggested that an elevated RR may indicate increased respiratory demand [13]. In the present study, a higher RR was associated with SD at 28 days after extubation. This finding suggests that respiratory status at the initial swallowing assessment may be related to later swallowing outcomes in patients with PED.

One possible explanation is impaired synchrony between respiration and swallowing. Swallowing normally occurs in coordination with respiration, and disruption of this pattern may increase aspiration risk [14,15]. In patients with PED, tachypnea may reflect respiratory stress that interferes with safe swallowing. Although the underlying mechanism was not directly evaluated in this study, the association between RR and SD should be interpreted cautiously.

These findings may have implications for ICU practice. Previous studies have reported that delayed speech-language pathology intervention after extubation is associated with poorer outcomes in patients with PED [4]. However, bedside indicators that help determine readiness for swallowing rehabilitation remain limited [2]. RR and duration of intubation may provide clinically accessible information when evaluating swallowing status after extubation.

Limitations

Several limitations of this study should be noted. First, because this was a retrospective study conducted at a single center, selection bias could not be excluded, and the generalizability of the findings may be limited. Second, referral for speech-language pathology assessment and decisions regarding oral intake training were based on clinical judgment rather than a standardized protocol, which may have introduced variability in patient management. Third, the effects of analgesics and sedatives on breathing and swallowing were not systematically evaluated. Baseline frailty and nutritional status were also not fully assessed, although these factors may influence swallowing recovery after critical illness. In addition, the mechanisms linking longer intubation duration to swallowing dysfunction, such as disuse of swallowing-related muscles, laryngeal injury, or prolonged critical illness, were not directly evaluated in this study. Finally, the sample size was relatively small, which limited the number of variables included in the multivariable analysis and increased the risk of overfitting. Therefore, the findings should be interpreted cautiously as exploratory associations.

## Conclusions

In conclusion, a higher RR and a longer duration of intubation were independently associated with SD at 28 days after extubation in ICU patients with PED. These findings suggest that respiratory status and intubation duration may help identify patients at risk of prolonged dysphagia after extubation. Additional large-scale prospective studies are required to better understand the mechanisms of swallowing impairment in critically ill patients and to validate the results of the present study.
